# Switch to faricimab after initial treatment with aflibercept in eyes with neovascular age-related macular degeneration

**DOI:** 10.1007/s10792-024-03297-1

**Published:** 2024-09-05

**Authors:** Aisha A. Aldhanhani, Ola A. Azzam, Sahar H. AlAli, Khaled G. Almasri, Shaikha H. Aljneibi, Francesco Pichi

**Affiliations:** 1 Cleveland Clinic Abu Dhabi, Eye Institute, Al Maryah Island, PO Box 112412, Abu Dhabi, United Arab Emirates; 2 Cleveland Clinic Lerner College of Medicine, Case Western Reserve University, Cleveland, OH USA

**Keywords:** Neovascular age-related macular degeneration, Aflibercept, Switch, Faricimab

## Abstract

**Purpose:**

To investigate the efficacy and outcomes of switching neovascular age-related macular degeneration (nAMD) patients from aflibercept to faricimab, focusing on visual acuity, retinal fluid management, and treatment intervals. The primary aim was to assess the early outcomes in nAMD patients refractory to aflibercept and explore faricimab’s potential as a longer-lasting therapeutic alternative.

**Methods:**

A single-center retrospective study was conducted on 50 refractory nAMD patients at Cleveland Clinic Abu Dhabi from September 2022–May 2023. Patients were switched from aflibercept to faricimab, having met specific criteria for refractory nAMD. The study analyzed best-corrected visual acuity (BCVA), central subfield thickness (CST), and fluid changes post-switch, using Optical Coherence Tomography (OCT).

**Results:**

After three faricimab injections, significant reductions in CST were observed, with a notable decrease in retinal fluid. The mean BCVA remained stable throughout the study period. Although there was a decrease in the maximum pigment epithelial detachment (PED) height, it was not statistically significant. Treatment intervals post-switch showed that the majority of patients maintained or extended their treatment intervals, with a significant proportion achieving resolution of intraretinal fluid (IRF) and subretinal fluid (SRF).

**Conclusions:**

Switching to faricimab from aflibercept in refractory nAMD patients led to significant improvements in retinal fluid management and CST, with stable BCVA outcomes. Faricimab presents a promising alternative for patients requiring frequent aflibercept injections, potentially offering a more manageable treatment regimen with extended dosing intervals. This study highlights the need for personalized therapeutic strategies in nAMD treatment, though further research is necessary to optimize treatment switches.

## Introduction

The primary aim of anti-VEGF therapies in treating neovascular age-related macular degeneration (nAMD) is to preserve and potentially enhance visual acuity. However, the necessity for repeated anti-VEGF injections imposes significant burdens on both patients and healthcare providers [1, 2]. Despite efforts to mitigate this with treat-and-extend approaches, a subset of patients continues to require frequent injections [3–5]. Given these challenges, especially for patients with refractory nAMD, the need for alternative therapeutic options is evident. Continual vision deterioration in patients undergoing anti-VEGF treatment can be attributed to various factors, including the disease’s natural progression, suboptimal response to active treatment, and diminishing efficacy over time [6]. A body of prospective and retrospective studies has shown promising outcomes when patients unresponsive to initial treatments were switched to alternative anti-VEGF therapies [7–9], often resulting in reduced macular fluid and modest visual acuity improvements.

The advent of faricimab, a novel FDA-approved bispecific monoclonal antibody targeting both angiopoietin-2 and VEGF-A, offers a new therapeutic direction [10]. By simultaneously neutralizing these pathways, faricimab aims to provide a more durable treatment outcome. Angiopoietin-2 plays a critical role in vascular stability, angiogenesis, and permeability [11], often enhancing VEGF-induced angiogenesis in both healthy and diseased states. Elevated Ang-2 levels have been observed in nAMD cases [12, 13], underscoring its potential as a therapeutic target. The TENAYA and LUCERNE phase III trials [10] have shown that faricimab, with dosing intervals extendable up to every 16 weeks, matches the efficacy of 8-weekly aflibercept injections, possibly due to faricimab’s higher molar anti-VEGF dosage [14]. Preliminary studies involving patients previously treated with anti-VEGF agents have yielded encouraging results with faricimab [15–17].

This study aims to assess the early outcomes of transitioning nAMD patients from their current anti-VEGF treatments to faricimab, exploring its efficacy and potential as a longer-lasting therapeutic alternative.

## Methods

This study was a single-center, retrospective examination of consecutive cases of refractory nAMD where patients were transitioned from aflibercept to faricimab treatments at Cleveland Clinic Abu Dhabi, spanning from September 2022–May 2023. Eight retina specialists oversaw the patient care. Adhering to the Declaration of Helsinki’s ethical standards, the Cleveland Clinic's institutional review board sanctioned this research. The study’s retrospective, de-identified nature negated the need for patient consent.

The criteria for considering nAMD cases as refractory included patients who required monthly aflibercept injections due to persistent subretinal fluid (SRF), intraretinal fluid (IRF), and/or fluid beneath the retinal pigment epithelium (sub-RPE). Additionally, patients must have completed the three monthly loading phase of aflibercept and one injection within the ten weeks prior to the switch. Specifically, prior to the treatment shift, each patient had received injections of aflibercept every 4 or 5 weeks (average 4.3 ± 0.3 weeks) for a minimum of three times.

After switching to faricimab, patients were treated based on a treat-and-extend regimen. Initially, patients received three monthly injections to stabilize the condition, followed by individualized treatment intervals based on clinical assessment. The treatment interval could be extended by 2–4 weeks if the patient showed no signs of disease activity, with a maximum interval of 16 weeks. Treatment schedules were either maintained or extended according to clinician discretion, allowing for a personalized approach based on each patient’s response.

The data collection from medical records included age, sex, relevant previous ocular history, such as the number of injections before the switch, any prior ocular surgeries, lens status, and best-corrected visual acuity (BCVA). Macular Optical Coherence Tomography (OCT) (Spectralis HRA, Heidelberg Engineering, Heidelberg, Germany) was performed at every visit, with both qualitative and quantitative assessments. Blind reviewers analyzed scans at the baseline and subsequent visits for the presence of SRF, IRF, and pigment epithelial detachment (PED) within the macular cube. Further evaluations of OCTs considered changes in retinal fluid as either improvement, stability, or deterioration. Quantitative data collected at each visit included the central subfield thickness (CST, automatically calculated by the Spectralis machine) and the height of PED from the RPE to Bruch’s membrane at the PED’s highest point, measured on the corresponding b-scan at each visit. The primary endpoint was the change in BCVA and CST after three faricimab injections from the baseline.

## Statistical analysis

Baseline demographics were assessed with descriptive statistical analysis. Conversion from Snellen to approximate ETDRS letters was performed using previously established formulas. Repeated measures analysis of variance with Bonferroni correction was used to assess differences in VA, CST, and maximum PED height. The statistical analyses were run on the open access R software (R Studio Version 1.1.383, R Project, www.r-project.org). Data are presented as the mean ± standard deviation, and statistical significance was based on a *P* < 0.05.

## Results

In this study, 50 eyes from 50 patients who were not responding to treatment for nAMD were selected based on specific eligibility criteria. The participants were monitored for an average of 24.1 ± 5.1 weeks. Initial patient data before beginning intravitreal faricimab injections is detailed in Table 1.

**Table 1:** Changes in best corrected visual acuity, central subfoveal thickness and sub-RPE fluid, in patients with neovascular AMD switched from aflibercept to faricimab

All (*n* = 50)			
	Baseline	1 month	*P* value
BCVA (logMAR)	0.76 ± 0.54	0.83 ± 0.31	0.99
CST (µm)	407.16 ± 144.69	399.3 ± 167.2	0.73
PED height (µm)	205.3 ± 107.5	201.5 ± 98.4	0.87

The group had an average age of 70.3 ± 9.73 years, predominantly male (34 individuals, 68%), of Arabic ethnicity (50 individuals, 100%), and most were pseudophakic (41 individuals, 82%). Prior to the treatment shift, each patient had received injections of aflibercept every 4 or 5 weeks (average 4.3 ± 0.3 weeks) for a minimum of three times. Before moving to faricimab, the average number of anti-VEGF injections received was 28.66 ± 11.8.

Upon receiving the first dose of faricimab, 50 out of 50 eyes (100%) presented some form of retinal fluid, either SRF (76%), IRF (50%), or sub-RPE fluid (34%). A comprehensive eye examination was performed a month after the first faricimab injection. One month into the faricimab treatment, 9.5% of eyes had no detectable fluid, 19.4% showed a decrease in fluid, 31.3% had unchanged fluid levels, and 24.5% experienced an increase in fluid levels. No significant changes were observed in BCVA, CST, and maximal PED height at the one-month mark (Table 1).

**Fig. 1:**
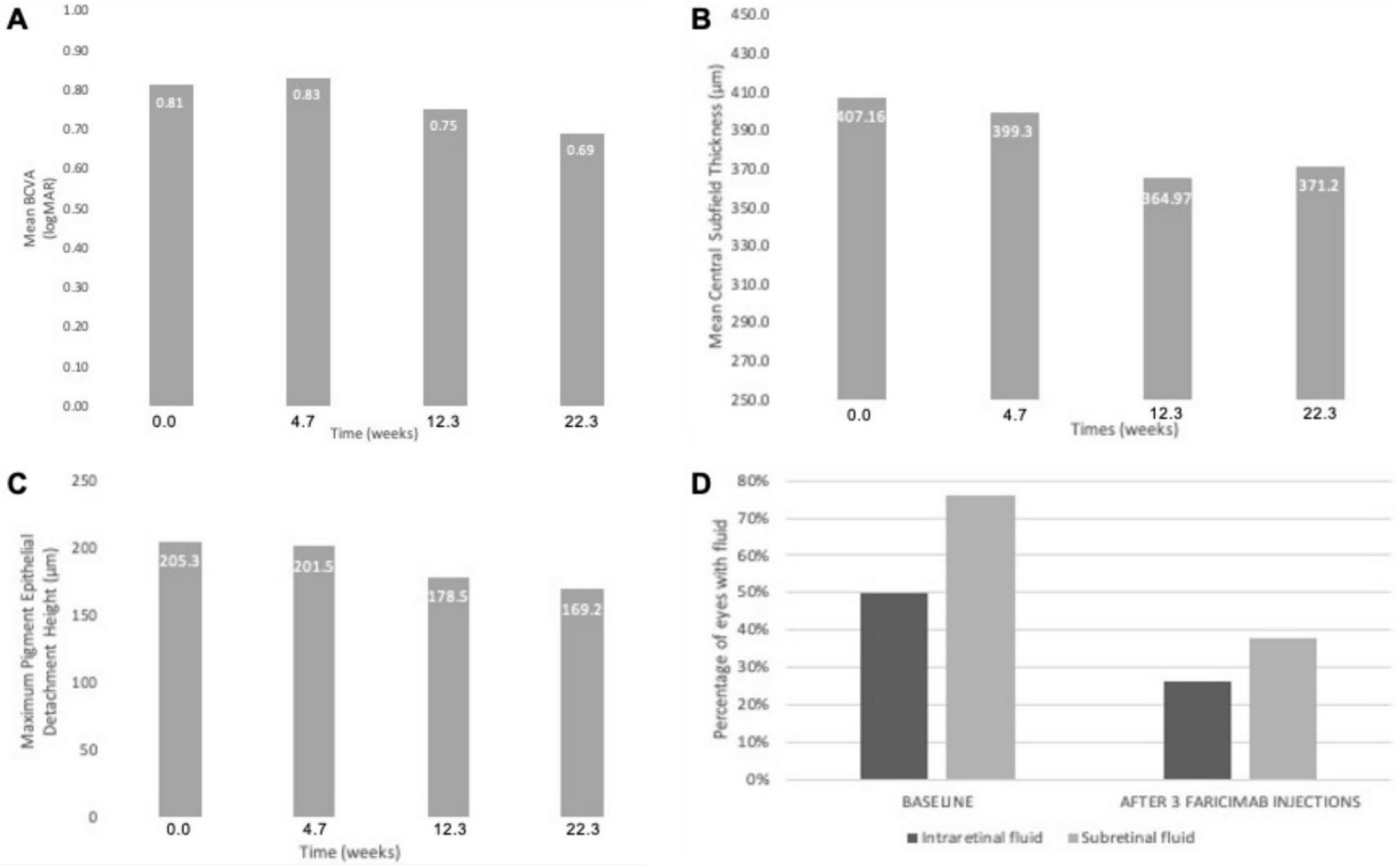
Comparison of best corrected visual acuity (**A**), central OCT subfield thickness (**B**), and pigment epithelium detachment (**C**), at switch from aflibercept to faricimab (time 0), after 1 intravitreal injection of faricimab (time 4.7 weeks), after 3 faricimab injections (time 12.3 weeks) and at the end of observation (time 22.3 weeks). Panel D shows decrease in intraretinal and subretinal fluid from baseline (switch to faricimab) till after 3 intravitreal injections of faricimab

However, after three intravitreal faricimab injections, there was a notable decrease in CST from 407.16 ± 144.69 µm to 364.97 ± 162.29 µm, indicating a significant improvement (*P* = 0.09) (Fig. 1B). Although there was a decrease in the maximum PED height following the third intravitreal faricimab injection (from 205.3 ± 107.5 µm to 178.5 ± 89.1 µm), this change did not reach statistical significance (*P* = 0.8) (Fig. 1C). The mean BCVA remained consistent throughout the treatment period (0.76 ± 0.54 logMAR pre-switch vs. 0.75 ± 0.56 logMAR after 3 faricimab injections, *P* = 0.45) (Fig. 1A).

The proportion of patients that had IRF after 3 intravitreal faricimab injections decreased to 26% (*P* < 0.01) as did the proportion of patients with SRF (38%, *P* < 0.01) (Fig. 1D). Of the eyes that had fluid before switching, 37 (74%) eyes had resolution of their IRF and 31 (62%) eyes demonstrated complete SRF resolution after 3 intravitreal faricimab injections. There were 14 (28%) patients that no longer had a PED present after 3 faricimab injections.

We analyzed the results according to the type of neovascularization (type 1 vs. type 2). While the small sample size limited the statistical power, we observed that both types of neovascularization showed improvements in retinal fluid management and CST reduction after switching to faricimab. However, type 1 neovascularization tended to show a slightly greater reduction in retinal fluid compared to type 2.

Treatment intervals did not significantly change post-switch, with the average interval between injections slightly altering from 5.4 weeks before switching to 6.1 weeks after the third intravitreal faricimab injection (*P* = 0.41).

## Discussion

The findings from this study enhance our comprehension of faricimab, a pioneering bispecific antibody targeting both VEGF and Ang-2 for the treatment of nAMD. We believe that eyes not responding to regular injections of aflibercept or anti-VEGF could greatly benefit from an innovative treatment with a more extended duration of effectiveness.

In our study group, we observed that faricimab effectively reduced retinal fluid in eyes previously treated with aflibercept, as evidenced by a notable average decrease in central subfield thickness (CST) by 42.19 µm. While the reduction in the highest point of pigment epithelial detachment (PED) height was not statistically significant, it decreased by 26.8 µm, alongside an increase in the percentage of patients who showed no IRF or subretinal fluid SRF by 18% after three intravitreal faricimab injections, all while maintaining stable BCVA. Remarkably, these improvements were achieved without the need for monthly loading doses for the majority of the patients (68% had treatment intervals of 6 weeks or longer), as treatment schedules were either maintained or extended according to clinician discretion.

The analysis of treatment outcomes based on the type of neovascularization, though limited by our sample size, indicated potential differences in response to faricimab. Type 1 neovascularization appeared to benefit slightly more in terms of retinal fluid reduction compared to type 2. Further studies with larger sample sizes are needed to confirm these observations and optimize treatment strategies based on neovascularization type.

Our participants were transitioned from treatment with aflibercept—a highly effective agent known for its affinity to VEGF-A, VEGF-B, and placental growth factor—to faricimab. Despite the proven benefits of aflibercept, particularly its success in improving OCT outcomes in 89–91% of patients with refractory nAMD within six months [18, 19], it necessitates monthly injections for a significant fraction of patients (18–48%) [20, 21]. Our findings suggest that faricimab, with its higher molar dose, presents a promising alternative for patients requiring frequent aflibercept injections. Faricimab’s bispecific nature allows it to target both VEGF-A and Angiopoietin-2, potentially leading to more durable treatment outcomes. While faricimab and aflibercept have comparable VEGF-A affinity, faricimab's molar dose of 6 mg/50 µl is higher compared to aflibercept’s 2 mg/50 µl, which may contribute to its prolonged efficacy and extended dosing intervals [10, 14]. This higher molar dose may enable faricimab to provide sustained therapeutic levels, thereby reducing the treatment burden on patients and improving compliance. Further studies are needed to confirm these preliminary findings and optimize treatment protocols for nAMD.

This is the third largest cohort of nAMD patients switched from aflibercept to faricimab that has been reported in the literature. With 110 eyes switched from aflibercept to faricimab, Szigiato et al. [17] reported the largest single-center cohort of nAMD patients switched to faricimab. Their results are similar to ours, with improved retinal anatomy after 3 injections of faricimab, despite no significant gains in BCVA. In addition, the majority of their patients (73%) had treatment intervals > 6 weeks and did not receive monthly loading doses after switching as most clinicians maintained the prior treatment interval.

Kataoka et al. [15] reported that 37.7% and 15.1% of eyes showed unchanged or worsened fluid levels at 1 month after switching from monthly aflibercept, while 41% of eyes were able to continue on faricimab treatment at 6 months, with successful extension of the treatment interval from monthly to bimonthly. However, the eyes that continued with faricimab injections up to 6 months showed significant reductions in CST at 1 month but not at the 6-month visit.

The real-world application of our study is underscored by our focus on patients already receiving aflibercept injections, reflecting a common clinical scenario. This approach provides valuable insights into the practical outcomes of switching anti-VEGF therapies. Notably, a certain percentage of eyes did not show fluid level improvements one month post-switch, indicating the potential need for an additional faricimab injection. Despite no significant improvement in visual acuity, the reduction in retinal fluid is a critical measure for clinicians, facilitating extended treatment intervals and thereby reducing the overall treatment and follow-up burden for patients.

Our study does present limitations, including its retrospective design, which may introduce selection bias and lacks control for potential regression toward the mean. Decisions regarding the switch to faricimab, treatment intervals, and discontinuation of therapy were left to the discretion of the treating specialists, adding variability to the study. Additionally, the exclusive focus on an Arabic population may limit the applicability of the findings to other demographic groups.

In summary, our research highlights the efficacy of faricimab as an alternative treatment for nAMD, especially for patients not responding adequately to frequent aflibercept injections. Switching to faricimab led to significant improvements in retinal fluid management and retinal thickness after three IFIs, maintaining similar treatment intervals and visual acuity outcomes as prior treatments. The study underscores the complexity of nAMD treatment and the need for tailored therapeutic approaches, though further research is necessary to identify predictors for successful treatment switches.

## Data Availability

No datasets were generated or analysed during the current study.

## References

[1] Rosenberg D et al (2023) Efficacy, safety, and treatment burden of treat-and-extend versus alternative anti-VEGF regimens for nAMD: a systematic review and meta-analysis. Eye (Lond) 1:6–16. 10.1038/s41433-022-02020-7 10.1038/s41433-022-02020-7 PMC9829919 35396574

[2] Wykoff CC et al (2017) Randomized trial of treat-and-extend versus monthly dosing for neovascular age-related macular degeneration: 2-year results of the TREX-AMD study. Ophthalmol Retina 4:314–321. 10.1016/j.oret.2016.12.004 10.1016/j.oret.2016.12.004 31047517

[3] Mitchell P et al (2021) Efficacy and safety of intravitreal aflibercept using a treat-and-extend regimen for neovascular age-related macular degeneration: The ARIES study: a randomized clinical trial. Retina 9:1911–1920. 10.1097/iae.0000000000003128 10.1097/IAE.0000000000003128 PMC8384251 33782365

[4] Tsunekawa Y et al (2021) Four-year outcome of aflibercept administration using a treat-and-extend regimen in eyes with recurrent neovascular age-related macular degeneration. Jpn J Ophthalmol 1:69–76. 10.1007/s10384-020-00783-8 10.1007/s10384-020-00783-8 33159611

[5] Yamamoto A et al (2017) One-year outcomes of a treat-and-extend regimen of aflibercept for exudative age-related macular degeneration. Ophthalmologica 3:139–144. 10.1159/000458538 10.1159/000458538 28259869

[6] Binder S (2012) Loss of reactivity in intravitreal anti-VEGF therapy: tachyphylaxis or tolerance? Br J Ophthalmol 1:1–2. 10.1136/bjophthalmol-2011-301236 10.1136/bjophthalmol-2011-301236 22157632

[7] Grewal DS et al (2014) Visual and anatomical outcomes following intravitreal aflibercept in eyes with recalcitrant neovascular age-related macular degeneration: 12-month results. Eye (Lond) 7:895–899. 10.1038/eye.2014.101 10.1038/eye.2014.101 PMC4094798 24833178

[8] Kawashima Y et al (2015) Effects of aflibercept for ranibizumab-resistant neovascular age-related macular degeneration and polypoidal choroidal vasculopathy. Graefes Arch Clin Exp Ophthalmol 9:1471–1477. 10.1007/s00417-014-2838-5 10.1007/s00417-014-2838-5 25391986

[9] Wykoff CC et al (2014) Aflibercept treatment for patients with exudative age-related macular degeneration who were incomplete responders to multiple ranibizumab injections (TURF trial). Br J Ophthalmol 7:951–955. 10.1136/bjophthalmol-2013-304736 10.1136/bjophthalmol-2013-304736 PMC4078682 24518078

[10] Heier JS et al (2022) Efficacy, durability, and safety of intravitreal faricimab up to every 16 weeks for neovascular age-related macular degeneration (TENAYA and LUCERNE): two randomised, double-masked, phase 3, non-inferiority trials. Lancet 10326:729–740. 10.1016/s0140-6736(22)00010-1 10.1016/S0140-6736(22)00010-1 35085502

[11] Heier JS et al (2021) The angiopoietin/tie pathway in retinal vascular diseases: a review. Retina 1:1–19. 10.1097/iae.0000000000003003 10.1097/IAE.0000000000003003 33136975

[12] Oshima Y et al (2004) Angiopoietin-2 enhances retinal vessel sensitivity to vascular endothelial growth factor. J Cell Physiol 3:412–417. 10.1002/jcp.10442 10.1002/jcp.10442 15095288

[13] Regula JT et al (2016) Targeting key angiogenic pathways with a bispecific CrossMAb optimized for neovascular eye diseases. EMBO Mol Med 11:1265–1288. 10.15252/emmm.201505889 10.15252/emmm.201505889 PMC5090659 27742718

[14] Sahni J et al (2019) Simultaneous inhibition of angiopoietin-2 and vascular endothelial growth factor-a with faricimab in diabetic macular edema: BOULEVARD phase 2 randomized trial. Ophthalmology 8:1155–1170. 10.1016/j.ophtha.2019.03.023 10.1016/j.ophtha.2019.03.023 30905643

[15] Kataoka K et al (2024) Six-month outcomes of switching from aflibercept to faricimab in refractory cases of neovascular age-related macular degeneration. Graefes Arch Clin Exp Ophthalmol 1:43–51. 10.1007/s00417-023-06222-x 10.1007/s00417-023-06222-x 37668741

[16] Leung EH et al (2023) Initial real-world experience with faricimab in treatment-resistant neovascular age-related macular degeneration. Clin Ophthalmol. 10.2147/opth.S409822 37181079 10.2147/OPTH.S409822 PMC10167970

[17] Szigiato A et al (2024) Short-term outcomes of faricimab in patients with neovascular age-related macular degeneration on prior anti-VEGF therapy. Ophthalmol Retina 1:10–17. 10.1016/j.oret.2023.08.018 10.1016/j.oret.2023.08.018 37673396

[18] Cho H et al (2013) Aflibercept for exudative AMD with persistent fluid on ranibizumab and/or bevacizumab. Br J Ophthalmol 8:1032–1035. 10.1136/bjophthalmol-2013-303344 10.1136/bjophthalmol-2013-303344 23766432

[19] Yonekawa Y et al (2013) Conversion to aflibercept for chronic refractory or recurrent neovascular age-related macular degeneration. Am J Ophthalmol 1:29-35.e2. 10.1016/j.ajo.2013.03.030 10.1016/j.ajo.2013.03.030 23668679

[20] Gillies MC et al (2019) Effect of ranibizumab and aflibercept on best-corrected visual acuity in treat-and-extend for neovascular age-related macular degeneration: a randomized clinical trial. JAMA Ophthalmol 4:372–379. 10.1001/jamaophthalmol.2018.6776 10.1001/jamaophthalmol.2018.6776 PMC6459213 30676617

[21] Maruko I et al (2020) Two-year outcomes of treat-and-extend intravitreal aflibercept for exudative age-related macular degeneration: a prospective study. Ophthalmol Retina 8:767–776. 10.1016/j.oret.2020.03.010 10.1016/j.oret.2020.03.010 32417356

